# Electroacupuncture Promotes Post-Stroke Functional Recovery via Enhancing Endogenous Neurogenesis in Mouse Focal Cerebral Ischemia

**DOI:** 10.1371/journal.pone.0090000

**Published:** 2014-02-24

**Authors:** Yu Ri Kim, Ha Neui Kim, Sung Min Ahn, Yung Hyun Choi, Hwa Kyoung Shin, Byung Tae Choi

**Affiliations:** 1 Department of Korean Medical Science, School of Korean Medicine, Pusan National University, Gyeongnam, Yangsan, Republic of Korea; 2 Division of Meridian and Structural Medicine, School of Korean Medicine, Pusan National University, Gyeongnam, Yangsan, Republic of Korea; 3 Department of Biochemistry, College of Oriental Medicine, Dongeui University, Busan, Yangjeong, Republic of Korea; University of South Florida, United States of America

## Abstract

To investigate the question of whether electroacupuncture (EA) promotes functional recovery via enhancement of proliferation and differentiation of neuronal stem cells (NSCs) in ischemic stroke, EA stimulation with 2 Hz was applied at bilateral acupoints to Baihui (GV20) and Dazhui (GV14) in middle cerebral artery occlusion (MCAO) mice. EA stimulation improved neuromotor function and cognitive ability after ischemic stroke. EA stimulation resulted in an increase in the number of proliferated cells, especially in the subventricular zone (SVZ) of the ipsilateral hemisphere. Although a very limited number of NSCs survived and differentiated into neurons or astrocytes, EA treatment resulted in a significant increase in the number of proliferative cells and differentiated cells in the hippocampus and SVZ of the ipsilateral hemisphere compared to MCAO mice. EA stimulation resulted in significantly increased mRNA expression of *brain-derived neurotrophic factor* (BDNF) and *vascular endothelial growth factor* (VEGF). Protein levels of these factors were confirmed in the ipsilateral hippocampus and SVZ by immunohistochemical and Western blotting analyses. Expression of phosphorylated phosphatidylinositol-3-kinase, BDNF, and VEGF-mediated down-stream were enhanced by EA stimulation in newly formed neuroblasts. These results indicate that EA treatment after ischemic stroke may promote post-stroke functional recovery by enhancement of proliferation and differentiation of NSCs via the BDNF and VEGF signaling pathway.

## Introduction

Persistent neurogenesis has been identified in two restricted regions, namely the subventricular zone (SVZ) of the lateral ventricle and the subgranular zone (SGZ) of the hippocampal dentate gyrus, in the adult mammalian brain, including humans, not only in developing ones [1]–[3]. Many stimuli, exogenously applied agents, and endogenous factors or states appear to regulate adult neurogenesis [4]. Factors including exercise, environmental enrichment, spatial learning, pregnancy, and stroke are associated with up-regulation of adult neurogenesis in these regions, whereas stress and aging are associated with its down-regulation [4], [5].

Stroke is a leading cause of long-term motor disabilities relying on rehabilitation therapy because there is no effective treatment except tissue type plasminogen activator during the first hours after a stroke [6]. However, this therapy can only be administered to a small percentage of patients, and there is no effective treatment for improvement of functional recovery in the post-ischemic phase [7], [8]. Functional recovery after stroke can potentially be induced by stimulation of endogenous neurogenesis [9]. After stroke, the brain maintains the potential for neuronal replacement by persistence of neurogenesis from the SVZ, the SGZ, and the neocortical layer, however, a very limited number of survival cells from newborn neuronal precursors have been identified [3], [10], [11].

Endogenous neural stem cells (NSCs) produce new neurons and possibly improve neurological impairments; therefore, they can provide potential therapeutic targets for stroke therapy [3], [7]. Appropriate therapeutic strategy may be developed for stroke, if the transient increase of NSCs proliferation and their maturation can be stimulated by any treatment [10], [11]. Electroacupuncture (EA), engrafted electric stimulation, is accepted as a common complementary therapy for treatment of stroke and post-stroke rehabilitation [12]. EA stimulation at a low frequency of 2 Hz induces differential regulation of more genes related to neurogenesis [13]. EA treatment may enhance cell proliferation and differentiation in the neurogenic area under post-ischemic conditions, which is associated with improved brain function.

However, EA studies have either been on adult animal models or have involved cell proliferation only in restricted areas without any further study [13]–[15]. The functional recovery and molecular mechanisms underlying the neurogenesis induced by EA stimulation in the brain remain obscure. Results showing that EA treatment can induce proliferation and differentiation of NSCs and then show a beneficial effect for neurorepair in stroke would provide evidence for its utility as a neurogenesis-stimulating therapy in stroke. Therefore, we hypothesized that EA treatment after ischemic stroke would have functional benefits via enhancement of neurogenesis and maturation of NSCs in the brain, which could be helpful in development of better therapeutic treatments for stroke. We selected a mouse model of cerebral ischemia-reperfusion injury and investigated the proliferation and maturation of NSCs with neurofunctional recovery by EA stimulation and cell survival-related factors and its down-stream pathways underlying adult neurogenesis.

## Materials and Methods

### Animal

**Figure 1:**
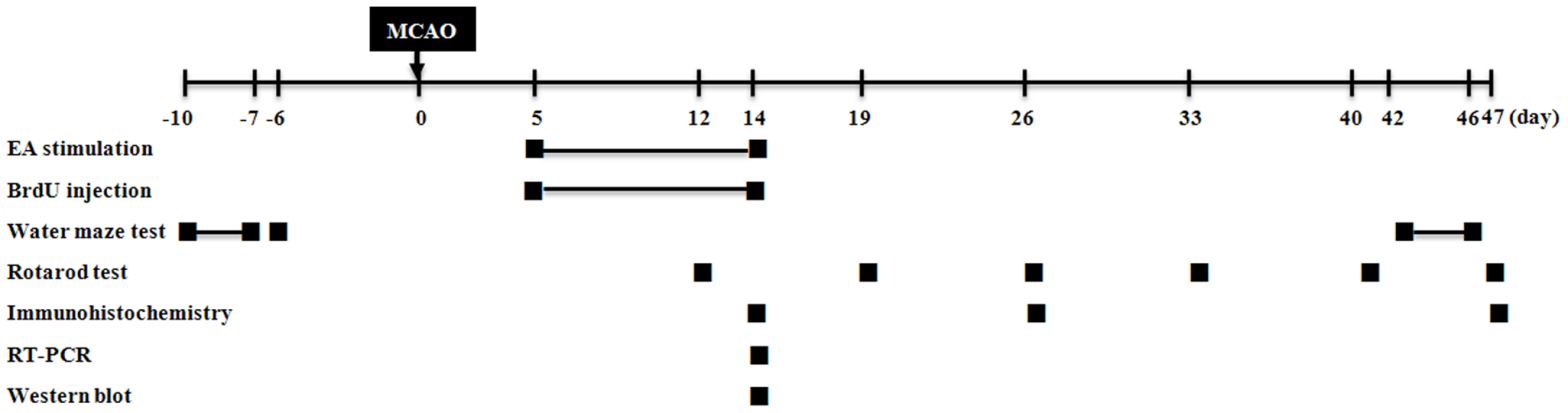
Schematic diagram for experimental procedures. All experiments were performed on the indicated day.

Male C57BL/6 mice, aged 10 weeks, were obtained from Dooyeol Biotech (Seoul, Korea). The mice were housed at 22°C under alternating 12 h cycles of dark and light, and were fed a commercial diet and allowed tap water ad libitum throughout the study. All experiments were approved by the Pusan National University Animal Care and Use Committee in accordance with the National Institutes of Health Guidelines. Each group consisted of six mice and all treatments were administered under isoflurane (Choongwae, Seoul, Korea) anesthesia, which was provided using a calibrated vaporizer (Midmark VIP3000, Orchad Park, OH, USA). Figure 1 shows the schematic diagram for experimental procedures and all experiments were performed on the indicated day.

### Focal Cerebral Ischemia

Focal cerebral ischemia was induced by occluding the middle cerebral artery (MCA) using the intraluminal filament technique. A fiber-optic probe was affixed to the skull over the middle cerebral artery for measurement of regional cerebral blood flow using a PeriFlux Laser Doppler System 5000 (Perimed, Stockholm, Sweden). Middle cerebral artery occlusion (MCAO) model was induced by a silicon-coated 4-0 monofilament in the internal carotid artery and the monofilament was advanced to occlude the MCA. The filament was withdrawn 40 min after occlusion and reperfusion was confirmed using laser Doppler.

### EA Stimulation

Under light isoflurane anesthesia, two bilateral stainless-steel 0.18 mm-diameter needles were inserted to a depth of approximately 2 mm at the acupoints corresponding to Baihui (GV20, the midpoint of the line connecting the apexes of both ears on the parietal bone) and Dazhui (GV14, the posterior midline and in the depression below the spinous process of the seventh cervical vertebra) in men, and were connected to a Grass S88 electrostimulator (Grass Instrument Co., West Warwick, RI, USA). EA treatment was administered with 2 Hz stimulation for 20 min and output voltage was set at 2 volts. EA was administered once per day for a successive 10 days from five days after MCAO. Subjects in the non-EA groups received only light isoflurane anesthesia for 20 min.

### Bromodeoxyuridine (BrdU) Labeling

BrdU (AbD Serotec, Oxford, UK) is a synthetic thymidine analog that becomes incorporated into a cell’s DNA when the cell is dividing during the S-phase of the cell cycle. For labeling of proliferating cells, all animals were injected with BrdU (50 mg/kg i.p.) once daily for 10 successive days during EA stimulation.

### Behavioral Assessment

Motor coordination and equilibrium were measured using a rotarod apparatus (Panlab S.L.U., Barcelona, Spain). After adaptation trials, each mouse was placed on the rotating rod for three trials per day at a speed of 20 rpm for 3 min and the time that an animal was able to hold itself on the rod was recorded. Acquisition training for the Morris water maze was performed on four consecutive days from 10 days to seven days before MCAO (five trials per day) and basal time was measured at six days before MCAO. The tank had a diameter of 100 cm and an altitude of 50 cm. The platform was placed 0.5 cm beneath the surface of the water. Each trial was performed for 90 s or until the mouse arrived on the platform. Results of the experiment were recorded using SMART 2.5.18 (Panlab S.L.U.).

### Immunohistochemistry

Mice anesthetized with isoflurane received intracardial perfusion with saline and then 4% paraformaldehyde in PBS. Brains were removed, post-fixed in the same fixative for 4 h at 4°C, and immersed in 30% sucrose for 48 h at 4°C for cryoprotection. Frozen 14 µm-thick sections were incubated for blocking with a blocking buffer (1X PBS/5% normal goat serum/0.3% triton X-100) for 1 h at room temperature. The sections were incubated with the following primary antibodies to BrdU (AbD Serotec, Oxford, UK), doublecortin (Dcx, Santa Cruz Biotechnology, Santa Cruz, CA, USA), neuronal nuclei (NeuN, Millipore Corporation, Billerica, MA, USA), glial fibrillary acidic protein (GFAP, Millipore Corporation), mature *brain-derived neurotrophic factor* (mBDNF, Abcam, Cambridge, UK), BDNF precursor (preBDNF, Santa cruz Biotechnology), *vascular endothelial growth factor* (VEGF, Santa cruz Biotechnology), phosphorylated phosphatidylinositol-3-kinase (pPI3K, Abcam, Cambridge, UK), and phosphorylated extracellular regulated kinase (pERK, Cell Signaling Technology, Danvers, MA, USA) overnight in PBS at 4°C. After washes with PBS, the sections were incubated with the fluorescent secondary antibody (Vector Laboratories, Inc., Burlingame, CA, USA) and DAPI (Invitrogen Corporation, Carlsbad, CA, USA) for 2 h and 30 min at room temperature in the dark, respectively, and then washed with PBS three times. Subsequently, slides were mounted in the mounting medium (Vector Laboratories, Inc.) and captured using a fluorescence microscope (Carl Zeiss Imager M1, Carl Zeiss, Inc., Gottingen, Germany) and a laser scanning confocal microscope (LSM 510, Carl Zeiss, Inc.).

### Reverse Transcription-polymerase Chain Reaction (RT-PCR)

Total RNA was prepared from brain tissue treated with TRIZOL reagent™ (Invitrogen, Paisley, UK) according to the manufacturer’s protocols. cDNA was synthesized using 2 µg of total RNA and oligodT_(18)_ primer with taq polymerase (Promega Corporation, Madison, WI, USA) in a 25 µl total reaction volume. Reverse transcription was performed by incubating the mixture at 37°C for 45 min, and the reaction was terminated at 95°C for 5 min. The following primers were used: 5′-AGGTGAGAAG-AGTGATGACCATCC-3′ (forward), and 5′-CAACATAAATCCACTATCTTCCCC-3′ (reverse) for BDNF; 5′-GCGGGCTGCCTCGCAGTC-3′ (forward) and 5′-TCACCGCCTTGGCTTGTCAC-3′ (reverse) for VEGF; 5′-GAGAGCCACATCGCCAGAG-3′ (forward) and 5′-TTTCGGGT-CAATGCACACTTG-3′ (reverse) for stromal cell-derived factor 1 (SDF-1); 5′-TGGACCGCAACAACGCCATCTATGAGAA-AACC-3′ (forward) and 5′-TGGAGCTGAAGCAATAGTTGGTATCCAGGGCT-3′ (reverse) for transforming growth factor beta 1 (TGF-β1); 5′-CTTCAGCATTCCCTTGACAC-3′ (forward) and 5′-AGCCTTCCTGCTGAGCA-CACA-3′ (reverse) for *nerve growth factor (NGF)*; 5′-CCCACGTTTCGCATGGTTC-3′ (forward) and 5′-TGGGCAGCTGAGGTTGTCAC-3′ (reverse) for glial cell-derived neurotrophic factor (GDNF); and 5′-ATGAGAAGGAGATC-ACTGC-3′ (forward) and 5′-CTGCGCAAGTTAGGTTTTGT-3′ (reverse) for β-actin. PCR products were then electrophoresed on 1% agarose gels and stained with ethidium bromide.

### Western Blot

Each brain tissue punch was washed in cold HEPES buffer and homogenized in lysis buffer [200 mM Tris (pH 8.0), 150 mM NaCl, 2 mM EDTA, 1 mM NaF, 1% NP40, 1 mM phenylmethanesulfonylfluoride, 1 mM Na_3_VO_4_, and protease inhibitor cocktail]. Equal amounts of proteins were then separated by 10% sodium dodecyl sulfate-polyacrylamide gel electrophoresis, followed by transfer of the resolved proteins to a nitrocellulose membrane (Whatman, Dassel, Germany). The membranes were incubated with the same primary antibody used in immunohistochemistry overnight at 4°C. Subsequently, membranes were incubated with secondary antibody. Actin was used as a loading control for all experiments. Quantification of immunoreactivity corresponding to the bands was performed by densitometric analysis using an ImageQuant LAS 4000 (Fujifilm, Tokyo, Japan).

### Data Analyses

All data are expressed as mean±SEM and were analyzed using the Sigmastat statistical program Version 11.2 (Systat Software, San Jose, CA, USA). Statistical analysis of data was performed using Student’s t-test when comparing two groups, or one-way ANOVA via Tukey’s post hoc comparison when comparing more than two groups. A *p*<0.05 was considered statistically significant.

## Results

### Effects of EA Treatment On Post-stroke Behavior

**Figure 2:**
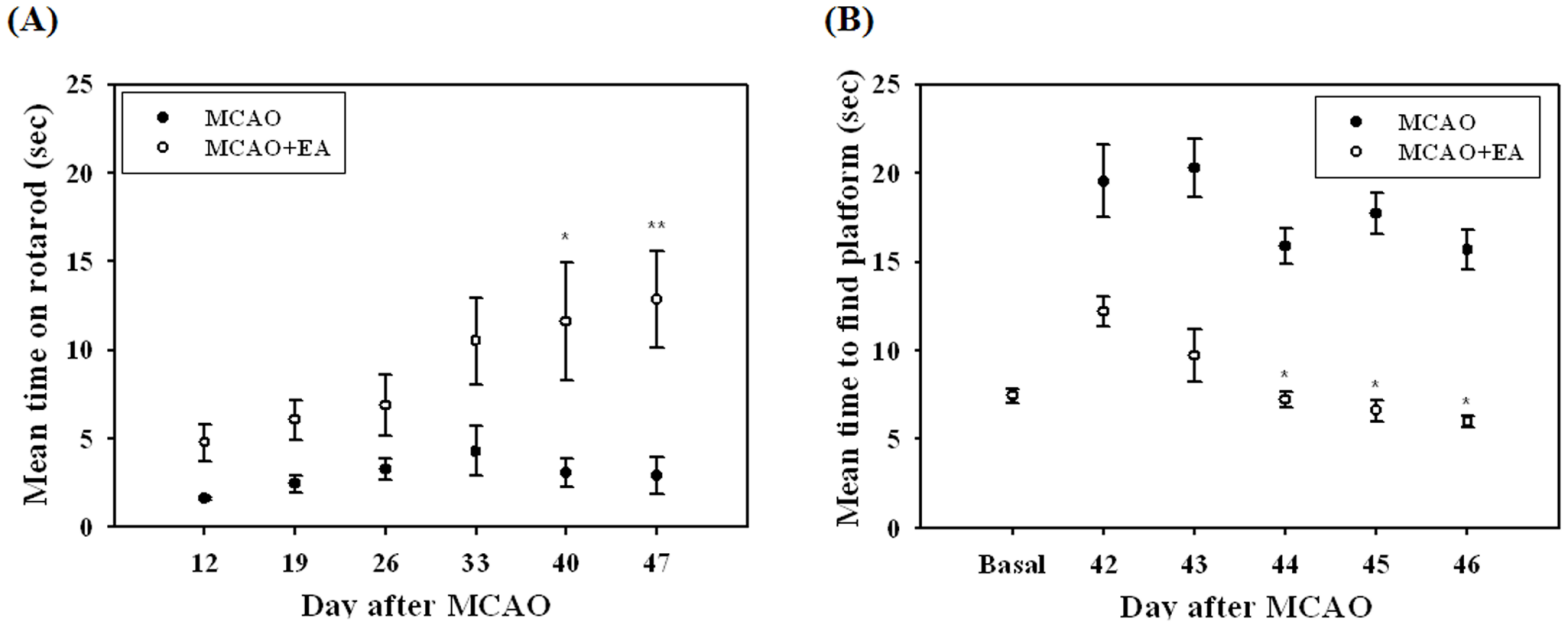
Behavioral assessment. Rotarod (A) and Morris water maze tests (B) on post-stroke behavior. EA treatment significantly improved neuromotor and cognitional function of MCAO mice at the late phase of the experiment. Mean±SEM. *P<0.05 and **P<0.01 versus MCAO mice.

To determine whether EA stimulation can improve the recovery of damaged neural function induced by MCAO, the rotarod and Morris water maze tests were applied and the mouse’s neuromotor and cognitive dysfunction was evaluated. No difference in the rotarod test of normal mice was observed between groups, however, EA-treated MCAO mice showed a significantly longer mean time compared with MCAO mice at 40 and 47 days after MCAO (Figure 2A). In the Morris water maze test, both MCAO and MCAO+EA mice took a longer time on average to find the platform than basal. However, compared with MCAO mice, EA-treated mice attained a significantly lower time from 44 to 46 days after MCAO (Figure 2B). These results suggest that EA treatment can induce beneficial effects for improvement of neuromotor and cognitional function in a focal cerebral ischemia model.

### Effects of EA Treatment On Neurogenesis in the Whole Brain

**Figure 3:**
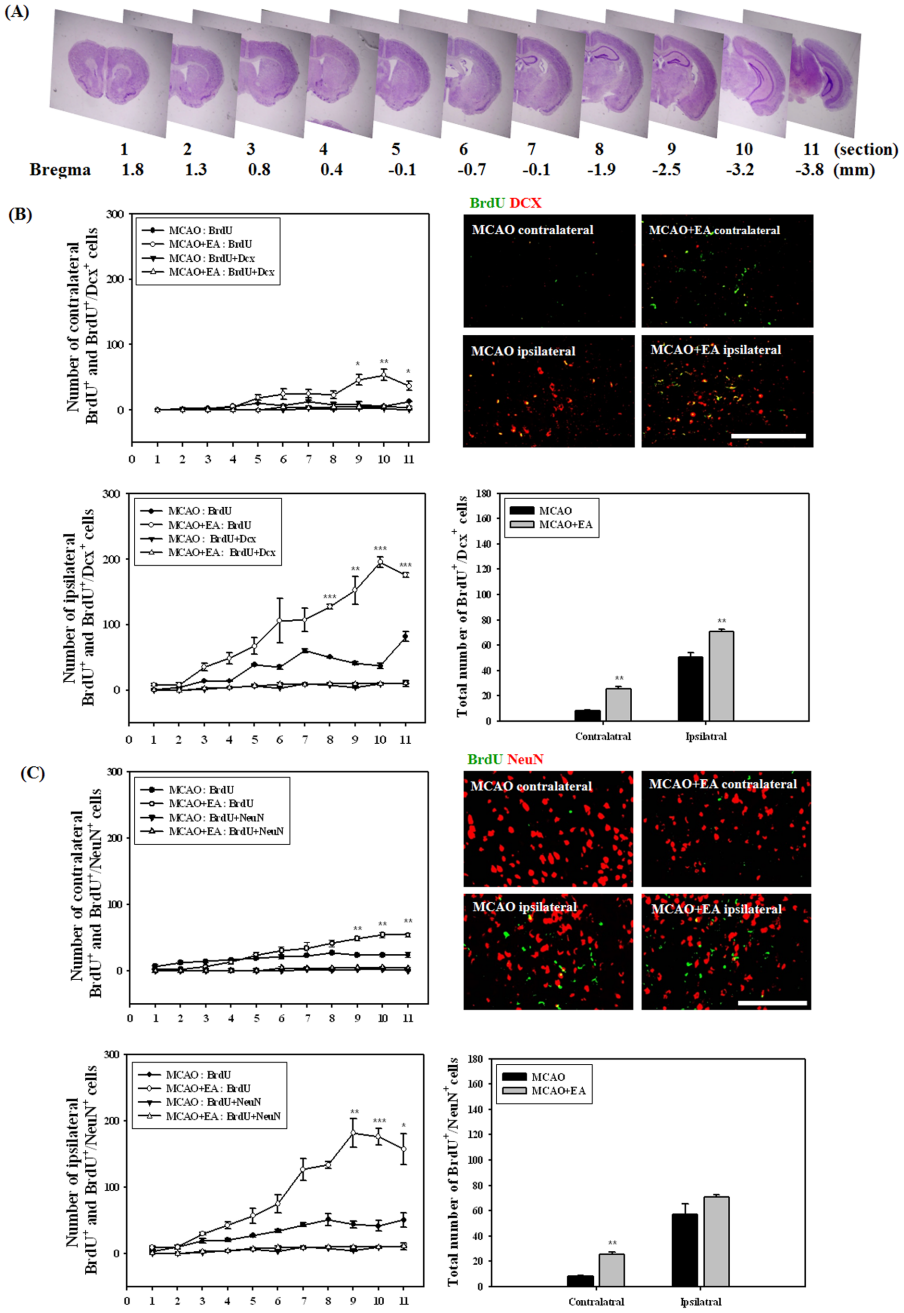
Effects of EA treatment on neurogenesis in the whole brain at 26 days after MCAO. Serial sections of the whole brain showing the regions for immunohistochemical analysis (A, cresyl violet stain) and its analysis for double-positive cells BrdU with neuroblast marker Dcx (B) or neuronal marker NeuN (C). Photomicrograph represents a typical result in the cortex. The number of proliferated NSCs indicated by BrdU positive was increased in both hemispheres of MCAO mice and EA treatment significantly increased the number of these cells in the posterior region of the brain. Total number of BrdU and Dcx or NeuN double-positive cells was significantly increased by EA stimulation. *P<0.05, **P<0.01 and ***P<0.001 versus MCAO mice. Scale bars = 100 µm.

To evaluate the effects of EA stimulation on endogenous NSCs, cells were identified by BrdU labeling and by a specific molecular marker such as Dcx and NeuN at 26 days after MCAO. At each 600 µm interval, 11 sections were taken for staining with cresyl violet or other molecular markers. We divided the whole brain into 11 coronal sections, as shown in Figure 3A, and then the total number of NSCs was evaluated by counting BrdU positive cell and double-positive cells for BrdU/Dcx or BrdU/NeuNs. The number of proliferated cells indicated by BrdU labeling was increased in both the ipsilateral and contralateral hemisphere of MCAO mice. EA stimulation resulted in a significant increase in the number of BrdU positive cells from the 8^th^ to 11^th^ coronal sections (from −1.9 mm to −3.8 mm to bregma) compared with MCAO mice (Figures 3B and 3C). Because it cannot be assumed that BrdU positive cells in the ischemic brain are newly born, we detected co-localization of BrdU with Dcx or NeuN, which is used routinely for identification of newly born neuroblasts and neurons. Compared with the total number of BrdU positive cells, the number of BrdU/Dcx or NeuN double-positive cells was very low, and no significant difference was observed throughout each of the coronal sections. However, the total number of BrdU with Dcx or NeuN positive cells in the whole brain was significantly increased by EA stimulation in the both contralateral and ipsilateral hemispheres and contralateral, respectively, (Figures 3B and 3C). These results demonstrated that EA treatment improves the division of NSCs after ischemic induction, however, a limited number of cells show beneficial effects through differentiation into neuronal cells.

### Effects of EA Treatment On Proliferation and Differentiation of NSCs

**Figure 4:**
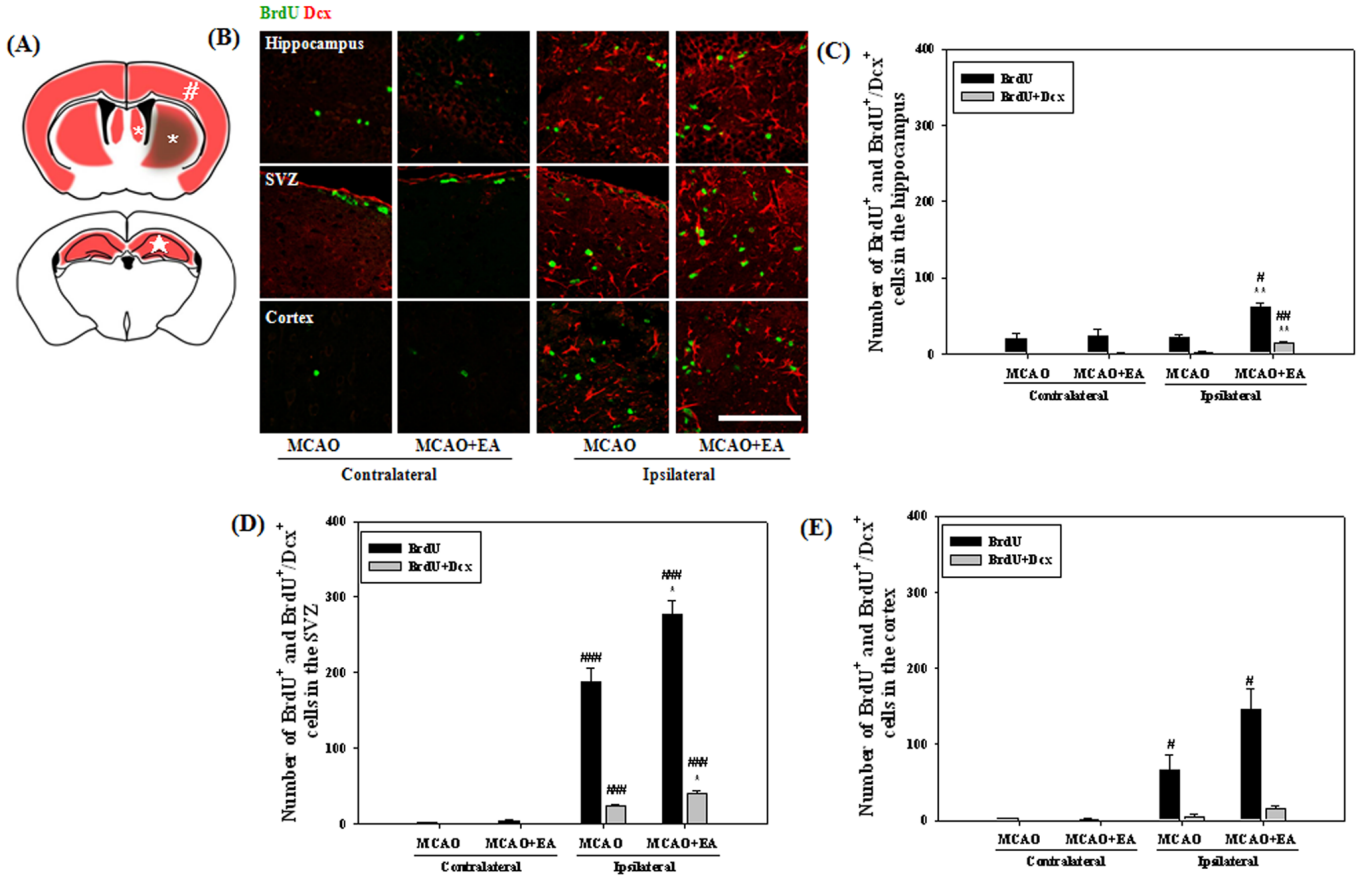
Effects of EA treatment on neurogenesis at 14 days after MCAO. Schematic diagram shows the regions of the hippocampus (★, in and around the hippocampus), SVZ (*, including the striatum) and cortex (#, the whole cortex) of the brain for immunohistochemical and further Western blot analysis (A). Photomicrograph (B) and its histogram for BrdU/Dcx double-positive cells in the hippocampus (C), SVZ (D) and cortex (E) of the brain. BrdU/Dcx double-positive cells were significantly increased by EA treatment in the hippocampus and SVZ of the ipsilateral hemisphere. ^#^ *P*<0.05, ^##^ *P*<0.01 and ^###^ *P*<0.001 versus the contralateral hemisphere; *P<0.05 and **P<0.01 versus MCAO mice. Scale bars = 100 µm.

**Figure 5:**
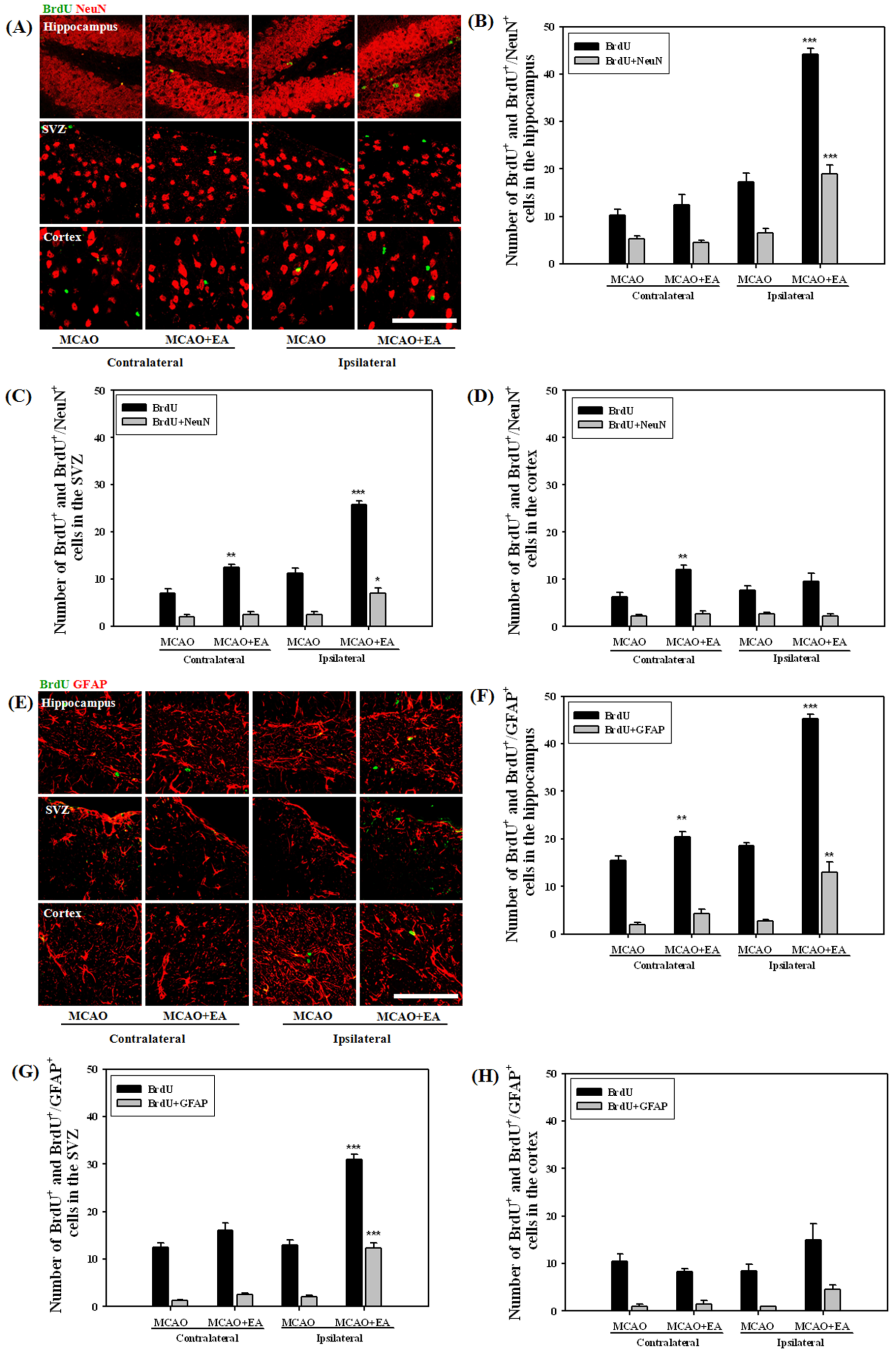
Effects of EA treatment on neurogenesis at 47 days after MCAO. Photomicrograph (B,E) and its histogram for BrdU/Dcx or NeuN double-positive cells in the hippocampus (B,F), SVZ (C,G) and cortex (D,H) of the brain. Both BrdU/NeuN and BrdU/GFAP double-positive cells were significantly increased by EA treatment in the ipsilateral hippocampus and SVZ. *P<0.05, **P<0.01 and ***P<0.001 versus MCAO mice. Scale bars = 100 µm.

To compare the effects of EA stimulation on proliferation and differentiation of NSCs, we selected two coronal sites, 3^rd^∼4^th^ (0.8∼0.4 mm to bregma) and 8^th^∼9^th^ (–1.9∼–2.5 mm to bregma) sections at 14 and 47 days after MCAO, and then counted each positive cell in the hippocampus, SVZ and cortex (Figure 4A). At 14 days after MCAO, the number of BrdU positive cells showed a significant increase in the ipsilateral hemisphere compared with the contralateral, especially in the SVZ. NeuN positive cells were very rarely detected on this day (data not shown). A very limited number of cells showed a BrdU/Dcx double positive reaction compared to proliferated cells, however, these cells were significantly increased by EA stimulation in the hippocampus and SVZ of the ipsilateral hemisphere (Figures 4B–E). At 47 days after MCAO, we identified differentiated neurons or astrocytes from NSCs. The majority of surviving cells were differentiated into neurons or astrocytes. EA stimulation resulted in a significant increase in the number of both BrdU/NeuN and BrdU/GFAP double-positive cells in the hippocampus and SVZ (Figure 5). These results suggest that EA treatment can increase the number of newly formed neuroblasts and enhance differentiation into neurons or astrocytes in the ipsilateral hippocampus and SVZ of MCAO mice.

### Effects of EA Treatment On Growth and Neurotrophic Factors

**Figure 6:**
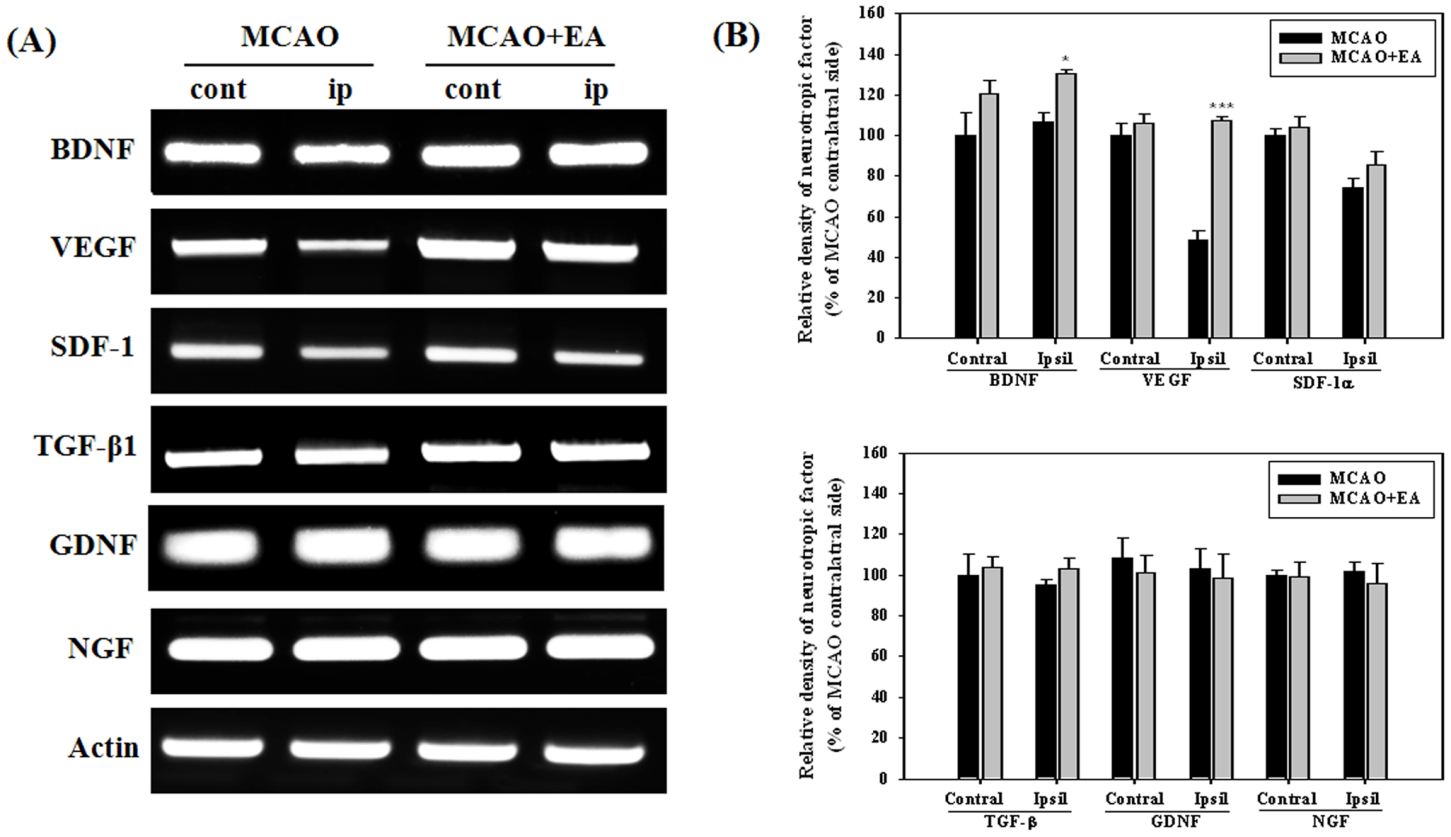
RT-PCR analysis for growth and neurotropic factors at 14 days after MCAO. Expression of each mRNA is expressed as a percentage of contralateral hemisphere of MCAO. Panel (A) its densitometric analysis (B). Panel represents a typical result from three independent experiments. EA treatment significantly increased the mRNA level of BDNF and VEGF in the ipsilateral hemisphere. *P<0.05 and ***P<0.001 versus MCAO mice.

**Figure 7:**
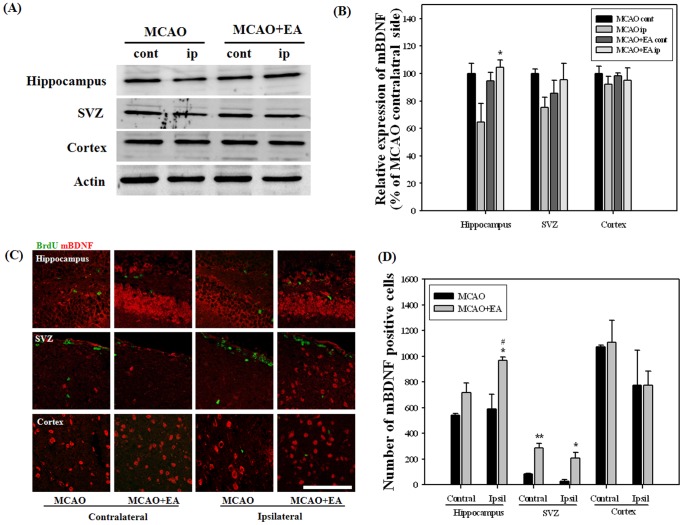
Western blot and immunohistochemical analysis for mBDNF at 14 days after MCAO. Western blot (A) and its densitometric analysis (B) showed that EA treatment significantly improved the expression of mBDNF in the ipsilateral hippocampus. Photomicrograph (C) and its histogram (D) showed that mBDNF positive cells were significantly increased by EA treatment in the ipsilateral hippocampus and SVZ. ^#^ *P*<0.05 versus the contralateral hemisphere; *P<0.05 and **P<0.01 versus MCAO mice. Scale bars = 100 µm.

**Figure 8:**
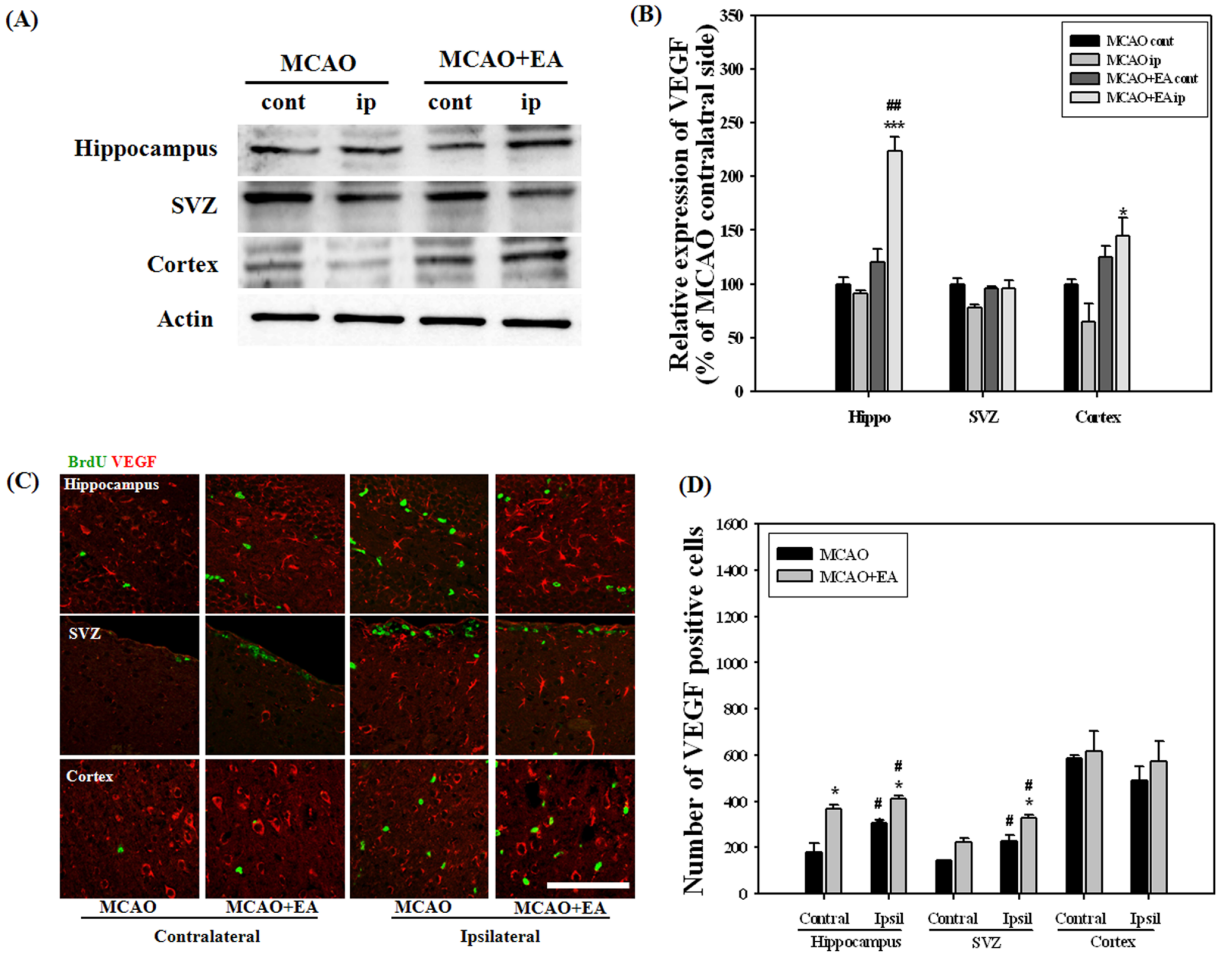
Western blot and immunohistochemical analysis for VEGF at 14 days after MCAO. Western blot (A) and its densitometric analysis (B) showed that EA treatment significantly improved the expression of VEGF in the ipsilateral hippocampus and cortex. Photomicrograph (C) and its histogram (D) showed that VEGF positive cells were significantly increased by EA treatment in the hippocampus and ipsilateral SVZ. ^#^ *P*<0.05 and ^##^ *P*<0.01 versus contralateral hemisphere; *P<0.05 and ***P<0.001 versus MCAO mice. Scale bars = 100 µm.

Growth and neurotrophic factors are potent regulators of adult neurogenesis. For screening of potential factors involved in EA stimulation, we performed RT-PCR analysis for the whole hemisphere at 14 days after MCAO, the last session of EA. BDNF and VEGF mRNA levels were significantly increased by EA stimulation in the ipsilateral hemisphere and no differences were observed in other factors (Figure 6). Immunohistochemistry and Western blot were performed in order to confirm BDNAF and VEGF expression by EA stimulation. Results of Western analysis showed that EA stimulation significantly improved expression of mBDNF and VEGF in the hippocampus and in both the hippocampus and cortex of the ipsilateral hemispheres, respectively. Immunohistochemical analysis showed that EA stimulation induced a significant increase in the number of mBDNF positive cells in the ipsilateral hippocampus and SVZ. The number of VEGF positive cells in the hippocampus and ipsilateral SVZ was also significantly increased by EA stimulation (Figures 7 and 8). These results suggest that BDNF and VEGF may play critical roles in EA-induced neurogenesis of MCAO mice.

### Effects of EA Treatment On Expression of pPI3K and pERK in Newly Born Cells

**Figure 9:**
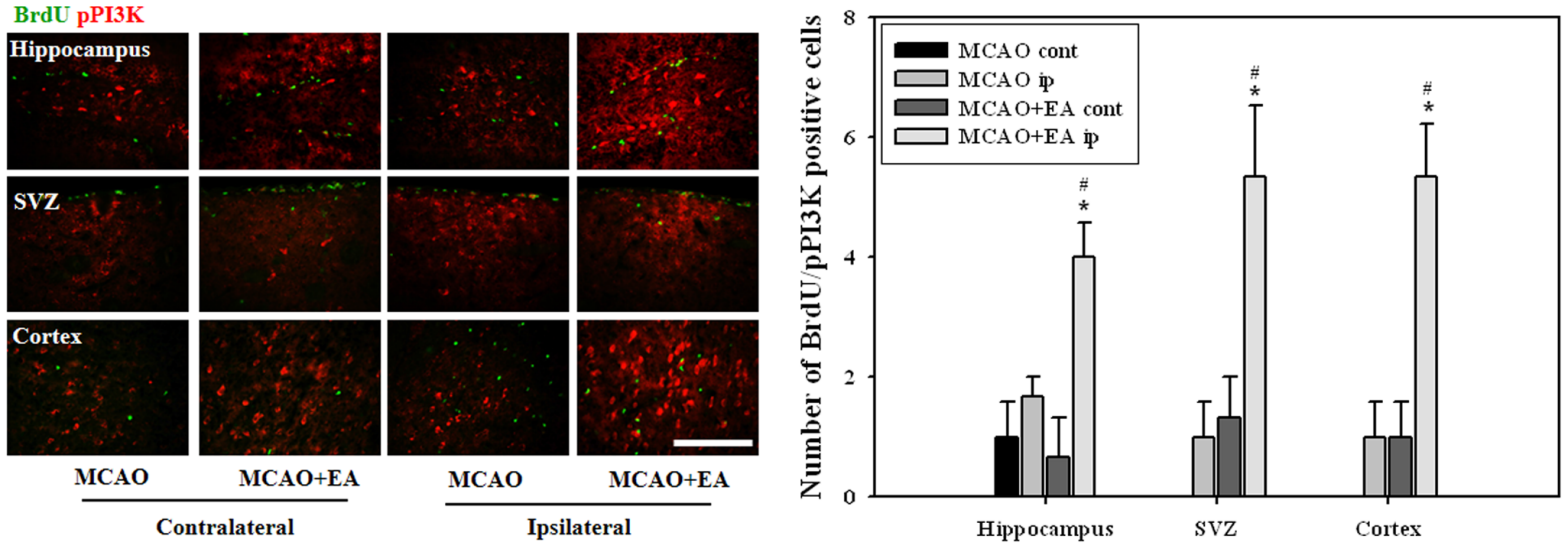
Immunohistochemical analysis for pPI3K in newborn cells at 14 days after MCAO. Number of pPI3K/BrdU double-positive cells was significantly increased by EA treatment in all regions examined. ^#^ *P*<0.05 versus contralateral hemisphere; *P<0.05 versus MCAO mice. Scale bars = 100 µm.

To determine whether down-stream signaling of BDNF and VEGF are involved in EA-induced neurogenesis, we observed the expression of phosphorylated PI3K and ERK in newly formed cells at 14 days after MCAO. Co-localized cells for BrdU with pPI3K were observed very rarely in both MCAO and MCAO+EA mice; however, cells for BrdU and pERK were barely detected. EA stimulation resulted in an increase in the number of pPI3K/BrdU double positive cells in all regions examined (Figure 9). These results suggest that EA stimulation may improve proliferation of NSCs against ischemic induction through activation of BDNF and VEGF mediated down-stream PI3K.

## Discussion

The adult brain is capable of replacing some lost neurons after stroke injury via three distinct steps, proliferation, migration, and differentiation of NSCs [16]. Marked cell proliferation and generation of neuroblasts has been reported in the SVZ following stroke injury; these cells migrate to the damaged area in the striatum [10], [11], [17]. New neurons from SVZ persist for a long term after stroke, at least several months [17]–[19]. Ultimately, neuroblasts that have migrated to injury sites show differentiation into a region-appropriate phenotype that becomes functionally integrated into neural networks for participation in brain repair and functional recovery after stroke [10], [17], [20], [21].

Although newly born cells can be supplied from several origins, including SVZ, SGZ, and the neocortical layer in the post-stroke brain, the number is too small for recovery of neurologic functions [3], [22], [23]. The fraction of dead striatal neurons that are replaced by newly born neurons at six weeks after insult is only about 0.2% [11]. These previous studies have provided comprehensive evidence indicating that strategies for neuronal replacement through adult endogenous neurogenesis may be of potential therapeutic value for stroke. However, simple proliferation of NSCs does not guarantee successful recovery from functional impairments. In order to become a therapeutic strategy for stroke, neurogenesis for capacity of self-repair has to be optimized for improvement of the poor survival of newborn neurons [11].

Positive effects of acupuncture are well known as a treatment for achievement of functional recovery after stroke [12], [24]. Thus, acupuncture signals that ascend mainly through the spinal ventrolateral funiculus to the brain may improve adult neurogenesis as a potent form of sensory stimulation [25]. EA treatment enhances stroke-induced striatal neurogenesis [26] and promotes neurological functional recovery via modulation of a key regulator of neurogenesis, retinoic acid [15]. The combination treatments of EA and NGF have a synergistic effect on cell proliferation and survival of NSCs, which is attributed to enhanced functional recovery [14].

Transient forebrain ischemia increases the number of NSCs and results in a peak level of proliferation at around 1–2 weeks after ischemic injury [3], [10], [11], [22]. Thus, we administered EA stimulation from five days to 14 days after MCAO on time showing a peak level of proliferated NSCs. We found that EA treatment after ischemic stroke resulted in improved neuronal function and induced proliferation and differentiation of NSCs.

We detected newborn cells when newborn neuroblasts expressed both specific marker, Dcx and NeuN (at 26 days after MCAO, three weeks after EA stimulation) [27]. EA treatment resulted in up-regulation of adult neurogenesis after stroke, however, in accordance with previous studies, very limited survival of newborn neuronal precursors was observed against the total number of BrdU positive proliferated cells [10], [11]. However, the increase in total numbers of BrdU/Dcx or NeuN double-positive cells indicates that EA stimulation may play beneficial roles in enhancement of proliferation and maturation of NSCs.

Thus, we compared proliferation and differentiation of NSCs in specific sites, including hippocampus, SVZ, and cortex at early and late phase after MCAO (14 days and 47 days after MCAO). The number of BrdU positive cells showed a significant increase in the SVZ of MCAO mice, compared with other sites, and EA treatment resulted in an increase in the number of these cells at early phase after MCAO. Neuroblast marker Dcx was observed in proliferated NSCs at early phase after MCAO, however, neuron and astrocyte markers, NeuN and GFAP, were detected at late phase. Fewer BrdU/NeuN and GFAP double-positive cells were detected in the SVZ and cortex at late phase after MCAO, compared with Brdu/Dcx positive cells at early phase, indicating loss or migration of NSCs during maturation. However, a larger number of differentiated cells was detected in the hippocampus, which may have caused migration of NSCs from a ventricular area caudal to the SVZ into the hippocampus in response to ischemia [28], namely the subcallosal zone (SCZ) and caudal extension of the SVZ [29]. NSCs in the neocortex of adult rats are also provided as a source of neurogenesis under ischemic conditions [23], however, no significant changes in the number of differentiated cells were observed by EA treatment.

Growth and neurotrophic factors have recently emerged as an important regulator of adult neurogenesis [4], [27]. Delivery of a neurotrophic factor can be a useful strategy for optimization of neurogenesis that improves the poor survival of newborn neurons. Acupuncture exerts therapeutic effects on animal models of pathologies through modulation of neurotrophin content in both the central nervous system and peripheral tissues [30].

Our results showed that BDNF and VEGF mRNA levels were significantly increased by EA treatment among considerable six factors considered as important regulators of adult neurogenesis. BDNF and angiogenesis factor VEGF are two important neurotrophic factors that have multiple effects on neurogenesis. BDNF and VEGF stimulate adult neurogenesis and enhance the appearance and migration of new neurons in the SVZ and dentate gyrus [31]–[34]. Post-ischemic intravenous BDNF treatment improves long-term functional neurological outcome for induction of neurogenesis [33], [35]. VEGF induces adult neurogenesis during exposure to an enriched environment or voluntary exercise [27] and reduces apoptosis after its infusion, suggesting a survival promoting effect of NSCs [36].

In examination of the brain by immunohistochemistry and Western blot, enhanced expression of mBDNF and VEGF occurred in parallel with the cellular proliferation and survival of NSCs. The current results imply potential roles of the BDNF and VEGF signaling pathway underlying the survival of NSCs. BDNF and VEGF mediate down-stream signaling cascades for survival of NSCs, such as PI3K/Akt or ERK pathways, via activation of the tyrosine kinase receptor (TrkB) and VEGF receptor 2, respectively [37]–[40]. After the last session of EA, an increase in the number of pPI3K positive cells was detected in BrdU positive cells.

In the current study, EA treatment improved neuronal function and induced proliferation and differentiation of NSCs through BDNF and VEGF signaling. The enhanced endogenous proliferation and maturation of NSCs in EA-treated mice may explain why functional recovery was observed after ischemic stroke. Consequently, EA stimulation induces proliferation of NSCs and promotes differentiation of proliferated cells into neurons or astrocytes, and provides the theoretical basis for a beneficial mechanism of EA treatment in post-ischemic stroke.
